# Investigation of the fungal community structures of imported wheat using high-throughput sequencing technology

**DOI:** 10.1371/journal.pone.0171894

**Published:** 2017-02-27

**Authors:** Yaqian Shi, Yinghui Cheng, Ying Wang, Guiming Zhang, Ruifang Gao, Caiyu Xiang, Jianjun Feng, Dingfeng Lou, Ying Liu

**Affiliations:** 1 Shenzhen Entry-Exit Inspection and Quarantine Bureau, Shenzhen, China; 2 Shenzhen Key Laboratory of Inspection Research & Development of Alien Pests, Shenzhen Scientific Academy of Inspection and Quarantine, Shenzhen, China; 3 Kunming University of Science and Technology, Kunming, China; Fujian Agriculture and Forestry University, CHINA

## Abstract

This study introduced the application of high-throughput sequencing techniques to the investigation of microbial diversity in the field of plant quarantine. It examined the microbial diversity of wheat imported into China, and established a bioinformatics database of wheat pathogens based on high-throughput sequencing results. This study analyzed the nuclear ribosomal internal transcribed spacer (ITS) region of fungi through Illumina Miseq sequencing to investigate the fungal communities of both seeds and sieve-through. A total of 758,129 fungal ITS sequences were obtained from ten samples collected from five batches of wheat imported from the USA. These sequences were classified into 2 different phyla, 15 classes, 33 orders, 41 families, or 78 genera, suggesting a high fungal diversity across samples. Apairwise analysis revealed that the diversity of the fungal community in the sieve-through is significantly higher than those in the seeds. Taxonomic analysis showed that at the class level, *Dothideomycetes* dominated in the seeds and *Sordariomycetes* dominated in the sieve-through. In all, this study revealed the fungal community composition in the seeds and sieve-through of the wheat, and identified key differences in the fungal community between the seeds and sieve-through.

## Introduction

China produces more wheat than any other country in the world. However, China’s supply of wheat cannot meet its huge domestic demand, due to increased consumption caused by population growth coupled with a lack of modern production technology. Therefore, it is a paradox that China is also one of the largest importers of. In fact, statistics show that from 2008 to 2014, China’s imports of wheat have grown at an average rate of 149% each year, from 3,90tons ($7.32M) to 288k tons ($932M). Nevertheless, careless wheat importation could cause significant damage to China’s agriculture production if alien pathogens invade China in the process and become established in China's ecological system. According to the latest report, there are over 200 wheat pathogens in the world, among which 25 can be transmitted through importation of the wheat (www.pestchina.com).

More and more evidence shows that crop seeds, like other parts of plants, are colonized by an epiphytic microbiota consisting of synergistic, commensal and potentially pathogenic microbes that play a critical role in plant health and susceptibility to disease[1–2]. The constituents of these microbial communities are likely to have functional relevance during plant growth, development and seed storage. More importantly, it was reported recently that the sieve-through of wheat contained a large fungal population which made the microbes of wheat pile increase and resulted in wheat deterioration [3–4]. The more microbes carried on the sieve-through, the more rapidly the storage quality of wheat changed. Despite their high biodiversity and critical ecological and economical roles, the fungal communities of wheat seed and sieve-through are still poorly studied relative to bacterial communities.

Wheat, along with maize and rice, provides 50% of human calories and is a critical food source in regions with rapid population growth including Asia, Africa and the Middle East. Productivity increases in wheat has slowed to 0.9% per year, yet need to increase to at least 1.5% per year by 2050 to avoid food price increases [5–6]. Healthy, high-quality seeds are critically important for the stability of the world’s food supply and the economic success of farmers. Host-associated microbes play an important role in the growth and development of the host [7–8]. However, the study of host-associated wheat microbes is mostly focused on the soil and root, and reports on microbes of wheat seeds are relatively rare.

Next generation sequencing is becoming an indispensable tool in fungal community ecology analysis and sequencing of the internal transcribed spacer (ITS) region of ribosomal fungal DNA allows identification down to the species level [9]. DNA sequencing of the fungal ITS region provides accurate, quantitative information about the diversity of a fungal community, which is not available from traditional culture-based analysis. While the potential of high-throughput sequencing is undisputed for studying complex fungal communities, our ability to understand the ecology of these communities has been hampered by insufficient sequencing depth and the high cost of 454 per sequencing. However, the Illumina Miseq platform provides sequencing at greater depth for a considerably lower price than per 454 sequencing, which promises a deeper characterization of fungal communities [10].

In this study, the fungal populations in the seeds and sieve-through of five imported USA wheat batches were investigated. These ten samples were subjected to Illumina Miseq sequencing of the internal transcribed spacer (ITS) region of the fungal nuclear ribosomal RNA gene. The primary objectives of this study were (1) to illuminate the fungal community composition in the seeds and sieve-through of wheat; and (2) to investigate differences between the fungal communities of seeds and sieve-through of wheat.

## Materials and methods

### Samples, DNA extraction and PCR amplification

Five batches of imported USA wheat were used for this study and each wheat sample was divided into seed and sieve-through using a 1.5mm Replacement Sieve. These five batches of wheat were imported from USA at different time. UW12 batch were imported at 2015/6, UW34 at 2015/7, UW56 at 2015/7, UW78 at 2016/1 and UW910 at 2016/2. Microbial DNA was extracted from seed and sieve-through using the E.Z.N.A. Fungal DNA Kit (Omega Bio-tek, Norcross, GA, U.S.) according to manufacturer’s protocols. The DNA concentration and quality were checked using a NanoDrop Spectrophotometer.

For sequencing, PCR amplification of the fungal ITS region was conducted with the ITS1 and ITS2 primers [11–12] using a 50 μL total volume with the following components: 25 μL PCR Master Mix (Roche, Indianapolis, IN, USA), 20 μL DNA-free water, 1.5 μL 10mM forward and reverse primers, and 2μL DNA template. PCR consisted of 95°C (5 min) for initial denaturation, followed by 36 cycles of 95°C (30 s) for dissociation, 55°C (30 s) for annealing, and 72°C (60 s) for each cycle extension, and finally 72°C (10 min) for final extension. The primers used in the reaction is 515F 5’-barcode- GTGCCAGCMGCCGCGG)-3’ and 907R 5’-CCGTCAATTCMTTTRAGTTT-3’, where barcode is an eight-base sequence unique to each sample.Unincorporated primers and PCR reagents were separated from PCR amplicons using the UltraClean 96 well PCR Clean-Up Kit (Mobio Laboratory, Carlsbad, CA, USA). The barcode is an eight-base sequence unique to each sample. PCR reactions were performed in triplicate in a 20-μL mixture containing 4 μL of 5× Fast Pfu buffer, 2 μL of 2.5 mM dNTPs, 0.4 μL of each primer (5 μM), 0.4 μL of Fast Pfu polymerase, and 10 ng of template DNA.

### Illumina MiSeq sequencing

Amplicons were extracted from 2% agarose gels and purified using the AxyPrep DNA Gel Extraction Kit (Axygen Biosciences, Union City, CA, U.S.) according to the manufacturer’s instructions and quantified using QuantiFluor™ -ST (Promega, U.S.). Purified amplicons were pooled in equimolar and paired-end sequenced (2 × 300) on an Illumina MiSeq platform according to the standard protocols. The raw reads were deposited into the NCBI Sequence Read Archive (SRA) database. (SRA accession: SRP077547)

### Processing and analyzing of sequencing data

Raw fastq files were demultiplexed, quality-filtered using QIIME (version 1.17) [13] with the following criteria: (i) The 300 bp reads were truncated at any site receiving an average quality score <20 over a 50 bps sliding window, discarding the truncated reads that were shorter than 50bp. (ii) exact barcode matching, 2 nucleotide mismatch in primer matching, and reads containing ambiguous characters were removed. (iii) Only sequences that overlap longer than 10 bp were assembled according to their overlap sequence. Reads which could not be assembled were discarded.

Operational Units (OTUs) were clustered with 97% similarity cutoff using UPARSE (version 7.1 http://drive5.com/uparse/) and chimeric sequences wereidentified and removed using UCHIME. The taxonomy ofeach 16S rRNA gene sequence was analyzed by RDP Classifier (http://rdp.cme.msu.edu/) against the unite 16S rRNA database using confidence threshold of 70%. The rarefaction analysis based onMothur v.1.21.1[14] was conducted to reveal the diversity indices, including the Chao’s species richness estimator (Chao), the abundance-based coverage estimator (ACE), and the Shannon-Weiner (Shannon) diversity indices. A representative sequence from each phylotype was aligned using the Python Nearest Alignment Space Termination (PyNAST) tool [13, 15] with a relaxed neighbor-joining tree built using FastTree [16]. A taxonomic identity for each representative phylotype was determined by a BLAST comparison against sequences within the unite database. To correct for sampling effort, we used a randomly selected subset of 4500 fungal sequences per sample to calculate the community composition. Principle Coordinate Analysis (PCoA) of weighted UniFrac distances as implemented in QIIME.

### Statistical analysis

The statistics analysis of the diversity difference between the seed and sieve-through was performed using the paired T-test. All analysis was performed using the SPSS 17.0. The significance level was set at 5%.

## Results

### Sequencing results and diversity indices

**Fig 1:**
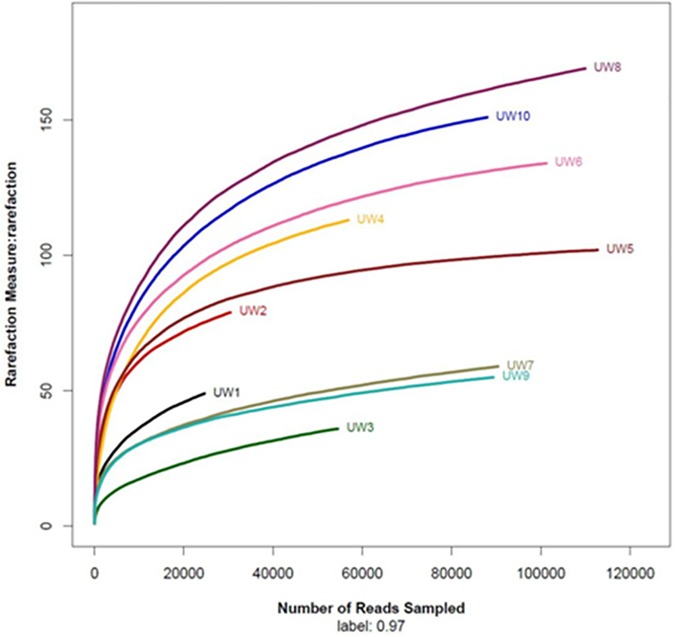
Rarefaction curves of the OTU number at 97% similarity for each sample

**Fig 2:**
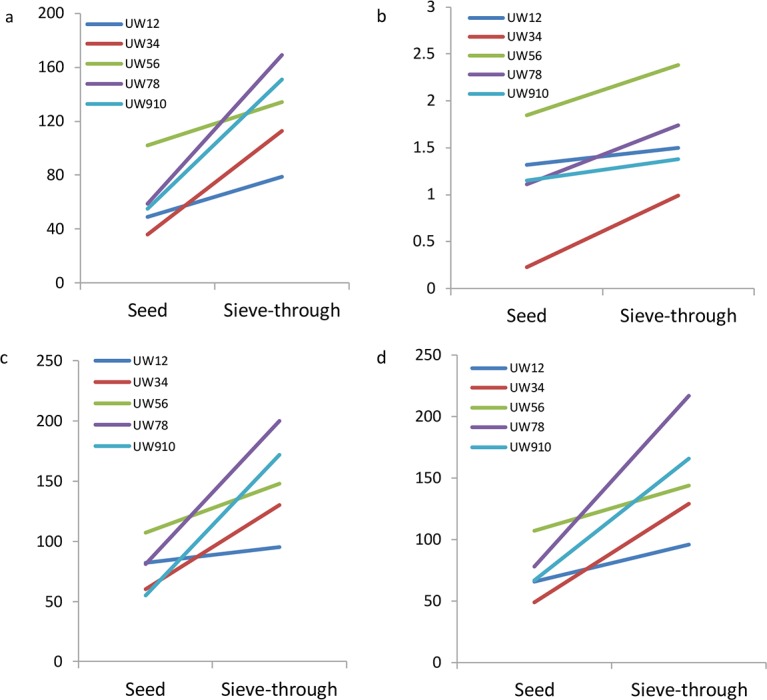
The pairwise comparison of the alpha diversity of seed and sieve-through of each wheat sample. a) OTU number, b) Shannon index c) ACE Estimator and d) Chao1 Estimator.

A total of 758,129 high-quality reads were obtained from ten samples through Illumina MiSeq sequencing analysis and 274 OTUs were observed at a 97% similarity. Each library contained 24,627 to 112,727 reads, with different phylogenetic OTUs ranging from 36 to 169 (Table 1). All rarefaction curves calculated with QIIME pipeline at 97% similarity tended to approach the saturation plateau, which indicated that the number of sequenced reads in each sample was reasonable, and the discovery of a high number of reads did not make a big contribution to the total number of OTUs. As shown in the rarefaction curve, there is a large variation in the total number of observed OTUs in different samples (Fig 1). Compared with the seeds, all samples from sieve-through have significantly higher OTU density based on the paired t-test (Fig 2). The OTU density of sample UW8 (sieve through group) is the highest and it is around 3 times of the OTU density of its matched seed (UW7). However, the total reads of UW7 and UW8 are comparable.

**Table 1:** MiSeq sequencing results and diversity estimates for each sample.

Type	Sample	Total sequences	Total OTUs^a^	ACE	Chao	Shannon	Coverage
	UW1	24627	49	82	66	1.32	0.99935
	UW3	54493	36	60	49	0.23	0.999725
Seed	UW5	112727	102	107	107	1.85	0.99992
	UW7	90403	59	81	78	1.11	0.999812
	UW9	89356	55	55	67	1.15	0.999832
	Mean	74321	60	77	73	1.132	0.999728
	UW2	30510	79	95	96	1.5	0.99941
	UW4	56852	113	130	129	0.99	0.999595
Sieve-through	UW6	101232	134	148	144	2.38	0.999802
	UW8	109946	169	200	217	1.74	0.999673
	UW10	87983	151	172	166	1.38	0.999693
	Mean	87983	129	149	150	1.598	0.999635

ACE: abundance-based coverage estimator, Chao: Chao’s species richness estimator, Shannon: Shannon-Weiner Index

Coverage is proportional to the nonsingleton phylotypes in all sequences.

^a^Species level, 97% similarity threshold used to define operational taxonomic units (OTUs).

Further, as shown in Table 1, the alpha diversity species richness (Chao), evenness (ACE), and Shannon index all confirmed the increasing diversity in the sieve through. The results of the paired t-test analysis of Chao, ACE and the Shannon diversity indices show significant differences, with P values of 0.018, 0.025 and 0.014, respectively (Fig 2). The average Chao and ACE of the sieve-through group were 150 and 149, respectively, approximately twice the values found in the seed group (Table 1). These results indicated large disparity existed between the two groups of samples for species richness and diversity of the fungal community.

### Taxonomic classification and abundance

**Fig 3:**
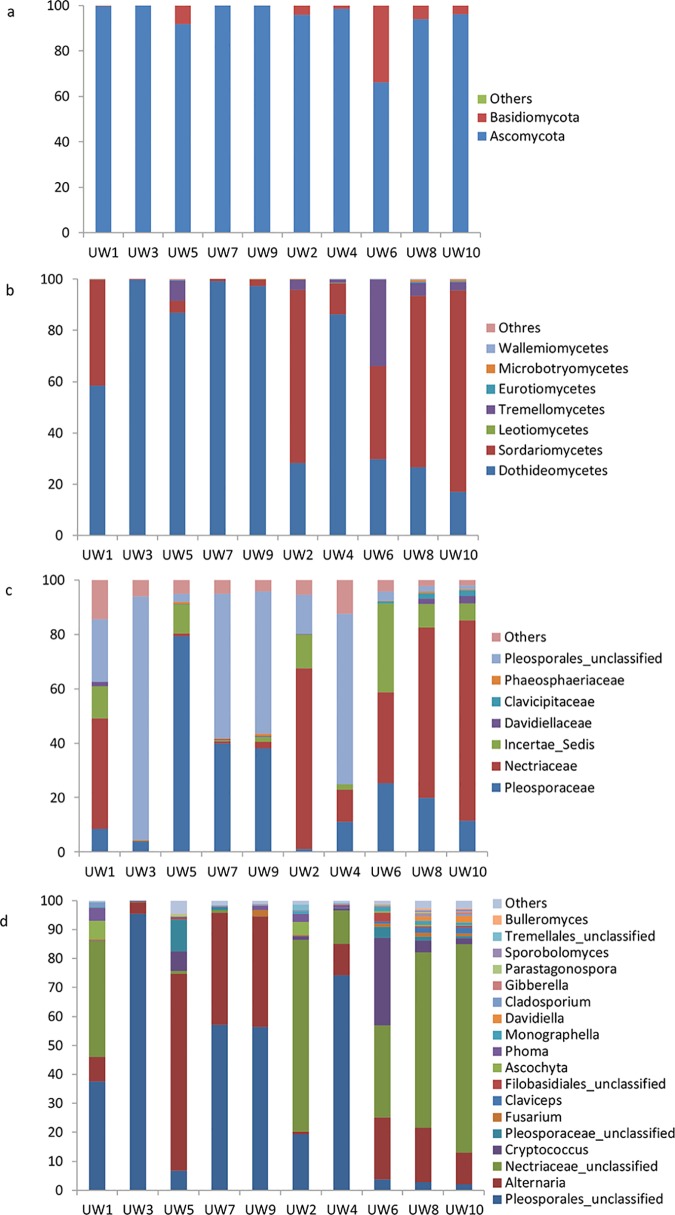
Relative abundances of the dominant fungal phyla (a), fungal classes (b), fungal family (c) and fungal genus (d) for each sample. Relative abundances are based on the proportional frequencies of the DNA sequences that could be classified.

Sequences that could not be classified into any known group were assigned to ‘unclassified’. The fungal OTUs were assigned into 2 different phyla, 15 classes, 33 orders, 41 families, or 78 genera. The dominant fungal phyla across all of these samples were *Ascomycota*, with relative abundances ranging from 66.2% to 99.8%. The *Basidiomycota* was the minor phyla with relative abundances ranging from 0.12% to 33.82%. No significant difference of the phyla abundance was found between seed and sieve-through (Fig 3A).

Further taxonomical classification at the class level revealed high levels of fungi belonging to *Dothideomycetes*, *Sordariomycetes*, *Microbotryomycetes*, *Eurotiomycetes*, *Tremellomycetes*, and *Leotiomycetes* in all of the soil samples (relative abundance > 1%), and these classes accounted for greater than 99% of the fungal sequences (Fig 3B). Another ten fungal classes, including *Wallemiomycetes*, *Microbotryomycetes*, *Exobasidiomycetes*, *Agaricomycetes*, and *Agaricostilbomycetes* etc. were less abundant (relative abundance < 1%) but still observed in several samples. *Dothideomycetes* and *Sordariomycetes* were the dominant fungal classes across these ten samples. Interestingly, the abundance of *Sordariomycetes* was significantly higher in the seed group compared with the sieve-through group, while the abundance of *Dothideomycetes* was significantly higher in the seed group (Fig 3B).

Taxonomical classification at the family level indicated that around 40 orders were detected. Among them, *Pleosporaceae*, *Nectriaceae and Pleosporales_unclassified* were dominant orders with relative abundances ranging from 1.07%-79.35%, 0.15%-87.05%, and 1.41%-89.48%, respectively. The orders of *Davidiellaceae*, *Clavicipitaceae*, *and Phaeosphaeriaceae* were also frequently observed, with mean relative mean of 0.75%, 0.47% and 0.34%. The abundance of *Nectriaceae* was significantly higher in sieve-through group compared with the seed group (Fig 3C).

A total of 78 genera were identified in these ten samples. The relative abundance of the genera Alternaria, unclassified genus (in the order Pleosporales) and unclassified genus (in the family Nectriaceae) were dominant in all samples. Among them, unclassified genus (in the order Pleosporales) was the most abundant (average abundance 35.52%) and occupied the dominant position in sample UW1, UW3, UW4, UW7 and UW9. The average abundances of Alternaria and unclassified genus (in the family Nectriaceae) were 22.05% and 28.40%, respectively (Fig 3D).

### Principal co-ordinates analysis

**Fig 4:**
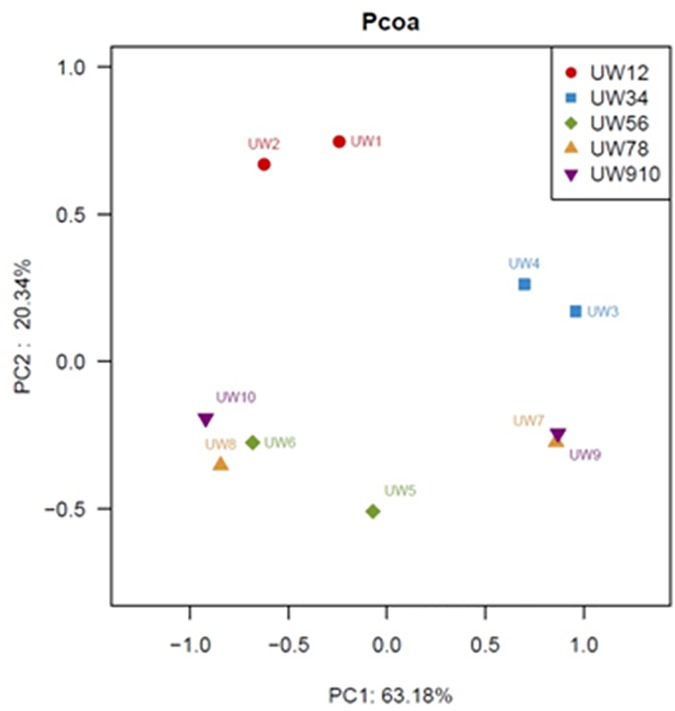
PCoA plot of weighted UniFrac distances of ten samples.

The differences in fungal communities between the samples were estimated using principal co-ordinates analysis. As shown in Fig 4, samples in UW12 and UW34 group cluster together, indicating the seed and sieve-through had similar fungal community. However, there was no clear community similarity in other three groups, indicating that the sieve-through may have completely different fungal structure from the seeds in the same wheat.

## Discussion

This study, using the high-throughput Illumina sequencing method, provided a detailed picture of fungal community variations at the phylum, class and family level between the seeds and sieve-through of wheat. The high diversity of fungal community in sieve-through suggested that sieve-through impurities should be separated as much as possible before wheat warehousing in order to reduce harm from the impurities to wheat quality during storage and increase the wheat storage stability.

Members of *Sordariomycetes* can grow in soil, dung, leaf litter, and decaying wood as decomposers, as well as being fungal parasites, and insect, human, and plant pathogens[17–18].The study showed that the Sordariomycetes dominated in the fungal population in the sieve-through and it may affect the healthy growth of the wheat if not removed from the seed.

This is the first study to analyze the fungal communities in the seed and sieve-through of imported USA wheat. 274 OTUs were identified from 758,129 high-quality reads in five samples of seed and five samples of sieve-through. The results of this study therefore expand our knowledge about the diversity and structure of microbial communities in seed and sieve-through of wheat. These results provide a system for understanding the microorganisms associated with seeds and sieve-through, and highlight the need for a thorough understanding of these microbial communities and their importance to the production and storage of healthy, high quality seeds.

This study used the most advanced sequencing technique to investigate the microbial diversity of imported USA wheat, thus providing the most comprehensive detection of pathogens at the DNA level and reducing the failure in detection of possible harmful microbes. It is valuable for the assessment of risk of imported Grain as well as for the establishment of policies for biocontrol and quarantine of imported Grain. It is also meaningful to ensure our agricultural production is safe as well as to boost international food exchanging and trading.

## References

[1] Critzer FJ, Doyle MP. Microbial ecology of foodborne pathogens associated with produce. Curr Opin Biotechnol. 2010;21(2):125–30. Epub 2010/02/13. 10.1016/j.copbio.2010.01.006 20149632

[2] Hashidoko Y. Ecochemical studies of interrelationships between epiphytic bacteria and host plants via secondary metabolites. Biosci Biotechnol Biochem. 2005;69(8):1427–41. Epub 2005/08/24. 10.1271/bbb.69.1427 16116269

[3] Jian F, Jayas DS, White ND. Movement and distribution of adult rusty grain beetle, Cryptolestes ferrugineus (Coleoptera: Laemophloeidae), in stored wheat in response to different temperature gradients and insect densities. J Econ Entomol. 2004;97(3):1148–58. Epub 2004/07/29. 15279304 10.1093/jee/97.3.1148

[4] Pardo E, Marin S, Ramos AJ, Sanchis V. Ecophysiology of ochratoxigenic Aspergillus ochraceus and Penicillium verrucosum isolates. Predictive models for fungal spoilage prevention—a review. Food Addit Contam. 2006;23(4):398–410. Epub 2006/03/21. 10.1080/02652030500376102 16546886

[5] Fan M, Lal R, Cao J, Qiao L, Su Y, Jiang R, et al Plant-based assessment of inherent soil productivity and contributions to China's cereal crop yield increase since 1980. PLoS One. 2013;8(9):e74617 Epub 2013/09/24. PubMed Central PMCID: PMC3776784. 10.1371/journal.pone.0074617 24058605 PMC3776784

[6] Zhang J, Yin B, Xie Y, Li J, Yang Z, Zhang G. Legume-Cereal Intercropping Improves Forage Yield, Quality and Degradability. PLoS One. 2015;10(12):e0144813 Epub 2015/12/18. PubMed Central PMCID: PMC4687681. 10.1371/journal.pone.0144813 26672990 PMC4687681

[7] Kim M, Yoon H, You YH, Kim YE, Woo JR, Seo Y, et al Metagenomic analysis of fungal communities inhabiting the fairy ring zone of Tricholoma matsutake. J Microbiol Biotechnol. 2013;23(10):1347–56. Epub 2013/08/21. 23949332 10.4014/jmb1306.06068

[8] Truyens S, Jambon I, Croes S, Janssen J, Weyens N, Mench M, et al The effect of long-term Cd and Ni exposure on seed endophytes of Agrostis capillaris and their potential application in phytoremediation of metal-contaminated soils. Int J Phytoremediation. 2014;16(7–12):643–59. Epub 2014/06/18. 10.1080/15226514.2013.837027 24933875

[9] Schoch CL, Seifert KA, Huhndorf S, Robert V, Spouge JL, Levesque CA, et al Nuclear ribosomal internal transcribed spacer (ITS) region as a universal DNA barcode marker for Fungi. Proc Natl Acad Sci U S A. 2012;109(16):6241–6. Epub 2012/03/29. PubMed Central PMCID: PMC3341068. 10.1073/pnas.1117018109 22454494 PMC3341068

[10] Yang C, Ji Y, Wang X, Yu DW. Testing three pipelines for 18S rDNA-based metabarcoding of soil faunal diversity. Sci China Life Sci. 2013;56(1):73–81. Epub 2012/12/28. 10.1007/s11427-012-4423-7 23269552

[11] Larena I, Salazar O, Gonzalez V, Julian MC, Rubio V. Design of a primer for ribosomal DNA internal transcribed spacer with enhanced specificity for ascomycetes. J Biotechnol. 1999;75(2–3):187–94. Epub 1999/12/20. 10553657 10.1016/s0168-1656(99)00154-6

[12] Manter DK, Vivanco JM. Use of the ITS primers, ITS1F and ITS4, to characterize fungal abundance and diversity in mixed-template samples by qPCR and length heterogeneity analysis. J Microbiol Methods. 2007;71(1):7–14. Epub 2007/08/09. 10.1016/j.mimet.2007.06.016 17683818

[13] Caporaso JG, Kuczynski J, Stombaugh J, Bittinger K, Bushman FD, Costello EK, et al QIIME allows analysis of high-throughput community sequencing data. Nat Methods. 2010;7(5):335–6. Epub 2010/04/13. PubMed Central PMCID: PMC3156573. 10.1038/nmeth.f.303 20383131 PMC3156573

[14] Schloss PD, Westcott SL, Ryabin T, Hall JR, Hartmann M, Hollister EB, et al Introducing mothur: open-source, platform-independent, community-supported software for describing and comparing microbial communities. Appl Environ Microbiol. 2009;75(23):7537–41. Epub 2009/10/06. PubMed Central PMCID: PMC2786419. 10.1128/AEM.01541-09 19801464 PMC2786419

[15] DeSantis TZ Jr., Hugenholtz P, Keller K, Brodie EL, Larsen N, Piceno YM, et al NAST: a multiple sequence alignment server for comparative analysis of 16S rRNA genes. Nucleic Acids Res. 2006;34(Web Server issue):W394–9. Epub 2006/07/18. PubMed Central PMCID: PMC1538769. 10.1093/nar/gkl244 16845035 PMC1538769

[16] Price MN, Dehal PS, Arkin AP. FastTree: computing large minimum evolution trees with profiles instead of a distance matrix. Mol Biol Evol. 2009;26(7):1641–50. Epub 2009/04/21. PubMed Central PMCID: PMC2693737. 10.1093/molbev/msp077 19377059 PMC2693737

[17] Berbee ML, Taylor JW. Two ascomycete classes based on fruiting-body characters and ribosomal DNA sequence. Mol Biol Evol. 1992;9(2):278–84. Epub 1992/03/01. 1560763 10.1093/oxfordjournals.molbev.a040719

[18] Celio GJ, Padamsee M, Dentinger BT, Bauer R, McLaughlin DJ. Assembling the Fungal Tree of Life: constructing the structural and biochemical database. Mycologia. 2006;98(6):850–9. Epub 2007/05/10. 17486962 10.3852/mycologia.98.6.850

