# Correction: Investigation of the fungal community structures of imported wheat using high-throughput sequencing technology

**DOI:** 10.1371/journal.pone.0197798

**Published:** 2018-05-17

**Authors:** The PLOS ONE Staff

The first author, Yaqian Shi, should not have been noted as the corresponding author. The second author, Yinghui Cheng, should be noted as the corresponding author. The publisher apologizes for this error.
